# Point-of-care Ultrasound to Evaluate Breast Pathology in the Emergency Department

**DOI:** 10.5811/westjem.2020.10.48008

**Published:** 2021-01-29

**Authors:** Josie Acuña, Cubby M. Pierre, Jacob Sorenson, Srikar Adhikari

**Affiliations:** * The University of Arizona, Department of Emergency Medicine, Tucson, Arizona; † The University of Arizona, College of Medicine, Tucson, Arizona

## Abstract

**Introduction:**

As physician-performed point-of-care ultrasound (POCUS) becomes more prevalent in the evaluation of patients presenting with various complaints in the emergency department (ED), one application that is significantly less used is breast ultrasound. This study evaluates the utility of POCUS for the assessment of patients with breast complaints who present to the ED and the impact of POCUS on medical decision-making and patient management in the ED.

**Methods:**

This was a retrospective review of ED patients presenting with breast symptoms who received a POCUS examination. An ED POCUS database was reviewed for breast POCUS examinations. We then reviewed electronic health records for demographic characteristics, history, physical examination findings, ED course, additional imaging studies, and impact of the POCUS study on patient care and disposition.

**Results:**

We included a total of 40 subjects (36 females, 4 males) in the final analysis. Most common presenting symptoms were breast pain (57.5%) and a palpable mass (37.5%). “Cobblestoning,” ie, dense bumpy appearance, was the most common finding on breast POCUS, seen in 50% of the patients. Simple fluid collections were found in 37.5% of patients.

**Conclusion:**

Our study findings illustrate the utility of POCUS in the evaluation of a variety of breast complaints in the ED.

## INTRODUCTION

Physician-performed point-of-care ultrasound (POCUS) has become increasingly more prevalent over recent years in the evaluation of patients presenting with various complaints in the emergency department (ED). One application significantly less used is breast ultrasound. It has been suggested that healthcare providers may be less confident in their ability to diagnose breast pathologies and concerned about potential litigation should an ominous pathology, such as malignancy, be missed. Breast complaints vary among many pathologies that can be unfamiliar to emergency physicians (EP).^1^ While prior studies have evaluated the use of ultrasound for patients who present to the ED with breast complaints, they are few in number and do not use physician-performed ultrasound examinations, but rather ultrasounds performed by sonographers and interpreted by radiologists.^2^ To our knowledge there are no studies to date that have evaluated the use of physician-performed POCUS for breast complaints in the ED.

Readily available in many EDs, POCUS makes the initial screening of breast complaints a viable option. It may be preferable to other imaging modalities due to absence of radiation, improved patient safety, real-time image acquisition, and relatively low cost.^3^–^7^ Point-of-care ultrasound allows for efficient and cost-effective decision-making in the care of ED patients. To our knowledge, no study to date has investigated the direct impact of POCUS on the medical decision-making of the EP in patients presenting with breast complaints. The purpose of this study was to evaluate the utility of POCUS for assessment of patients presenting to the ED with breast complaints and the impact of breast POCUS on medical decision-making and patient management in the ED.

## METHODS

### Study Design and Study Setting

We performed a a retrospective review of ED patients presenting with a breast complaint who received a POCUS over approximately a five-year period from November 27, 2014–December 29, 2019. This study includes breast POCUS examinations performed at two urban academic EDs totaling approximately 110,000 patient visits per year. Both EDs have an Accreditation Council for Graduate Medical Education (ACGME)-accredited emergency medicine (EM) residency program. One ED has an additional five-year combined EM/pediatrics residency program and an emergency ultrasound fellowship-training program. The residents receive emergency ultrasound training per ACGME guidelines. The attending physicians completed EM residency training and are board certified in EM. The attending physicians had been credentialed in superficial ultrasound.

Hospital-based credentialing in POCUS is available for ED attending physicians at both sites and was derived from the American College of Emergency Physicians ultrasound guidelines. Credentialing at these institutions required that physicians performed a minimum of 25 superficial ultrasounds. A specific ED POCUS protocol for evaluating breast pathology was not followed. Rather, when performing a POCUS examination of the breast, physicians took a similar approach as other superficial examinations. A high-frequency linear transducer was used for all studies. The entirety of the breast was examined, with more extensive imaging taking place at the area of concern. In cases where a suspected fluid collection, mass, or other concerning structure was identified, color Doppler was often used. In some cases, images of the contralateral breast were obtained for comparison.

The POCUS examinations included in this study were performed by both EM residents and attending physicians. All POCUS examinations were archived in the web-based workflow solutions database, Qpath (Q-path, Telexy Healthcare, Maple Ridge, BC, Canada), and quality assurance of all ultrasounds were performed by either emergency ultrasound fellows or emergency ultrasound fellowship-trained EPs. This database stores all POCUS examinations performed at both EDs, including interpretation reports detailing indications, findings, and final diagnoses that accompany each POCUS examination. Institutional board review approval was obtained for this study.

### Study population/inclusion criteria

We included patients in the study if they had received a breast POCUS examination in the ED and it was saved in the Qpath database. Patients received the POCUS when a credentialed EP was on duty.

**Population Health Research Capsule**

What do we already know about this issue?

*Point-of-care ultrasound (POCUS) is used in a variety of applications; its role in the evaluation of breast pathology has been less explored*.

What was the research question?

*Our goal was to evaluate the utility of POCUS for breast complaints in the emergency department*.

What was the major finding of the study?

*Emergency physicians are able to use POCUS to evaluate a variety of breast complaints*.

How does this improve population health?

*POCUS may impact management of breast pathology, aid in performance of procedures and treatment, and affect disposition*.

### Study Protocol

The Qpath database was initially queried for eligible subjects who received breast POCUS examinations followed by an electronic medical record review. A trained chart abstractor performed the chart review using a standardized data extraction form. The data extraction form included information about demographic characteristics, history, physical examination findings, ED course, POCUS findings, additional imaging studies, impact of breast POCUS on patient management in ED, disposition, and repeat visits to ED. Impact of breast POCUS on patient management was defined as the emergency provider’s decision to perform invasive procedures, order further imaging, request consultation, order antibiotics, and decision to admit or discharge the patient.

### Statistical Analysis

We used descriptive statistics to summarize the data. Continuous data were presented as means with standard deviations, and dichotomous and nominal data were presented as percentage frequency of occurrence.

## RESULTS

We included a total of 40 subjects (36 females, 4 males) and 40 breast POCUS studies in the final analysis. The mean age was 35.9 ±14.9 years (range 0–61). Pain was the most common presenting symptom (57.5%) followed by a palpable mass (37.5%). Presenting symptoms are summarized in Table 1. Patient characteristics were recorded and are summarized in Table 2. Half of the patients were found to have a history of skin/soft tissue infections, and 42.5% had a surgical history. The remaining characteristics documented made up a very small minority of the patients. The POCUS findings as reported by the EPs are listed in Table 3.

Of the 40 studies performed, the use of POCUS was documented in the medical decision-making section of the patient’s note in 38 (95%) of the cases. Six out of 40 POCUS studies were followed up by additional diagnostic imaging in the ED. Five were followed up by a dedicated breast US from radiology, and one was followed up by a chest radiograph. Of the patients who received a breast POCUS examination, 27 (67.5%) received antibiotics in the ED. Eleven patients received a surgery consult, and only three (27.3%) of these patients required additional imaging while in the ED. Thirteen patients underwent a needle aspiration in the ED as a result of POCUS examination findings that documented a fluid collection suspicious for an abscess. All of the procedures were documented as successful, confirming the presence of an abscess. None of the patients who had an ED procedure required additional imaging. Four of the patients who received a POCUS study in the ED required a procedure in the operating room.

With regard to patient disposition, 10 (25%) of the patients were admitted. Of the 30 patients who were discharged from the ED, 18 (60%) were sent home with a prescription for antibiotics. Seven patients had a repeat ED visit within three weeks of their visit. Fifteen patients had a documented follow-up within two weeks of their ED visit. Fourteen of these visits were to either breast clinic or oncology and one visit was with a primary care provider. The diagnoses made in the ED are summarized in Table 4. In some cases, patients received more than one diagnosis.

## DISCUSSION

The importance of access to breast ultrasound in the ED has been described.^8^,^9^ It is not uncommon for patients with breast pathology to present to the ED for an initial evaluation. The differential diagnosis for breast complaints is extensive, from trauma to infection and malignancy.^1^,^10^ Prior literature has shown that breast pain is one of the most common breast complaints.^11^,^12^ This is consistent with our study in patients presenting to the ED where a majority of the patients (57.5%) reported a complaint of breast pain, followed by patients presenting for evaluation of a palpable breast mass. While POCUS provides a potential answer for evaluating these patients, no prior literature exists on the use of this modality to evaluate breast complaints in the ED. For other applications, POCUS has already been found to play a critical role in screening for pathology and has several advantages over other imaging modalities. It is performed rapidly at the patient’s bedside by the treating clinician and is relatively inexpensive. Additionally, POCUS can also direct healthcare providers to more appropriate imaging modalities and consultations, as was demonstrated in our study.

One of the most common uses of breast ultrasound is to identify a drainable fluid collection when infection with abscess is being considered. This is of utmost importance for patients who present to the ED, as breast abscesses are generally considered to be a diagnosis that requires prompt intervention and treatment^8^,^13^ In our study, the majority of patients (57.5%) had either a simple or complex fluid collection found on POCUS. Of these patients, 65.2% received a final diagnosis of a breast abscess. These patients went on to undergo a procedure in the ED, and many were started on a course of antibiotics.

**Image 1:**
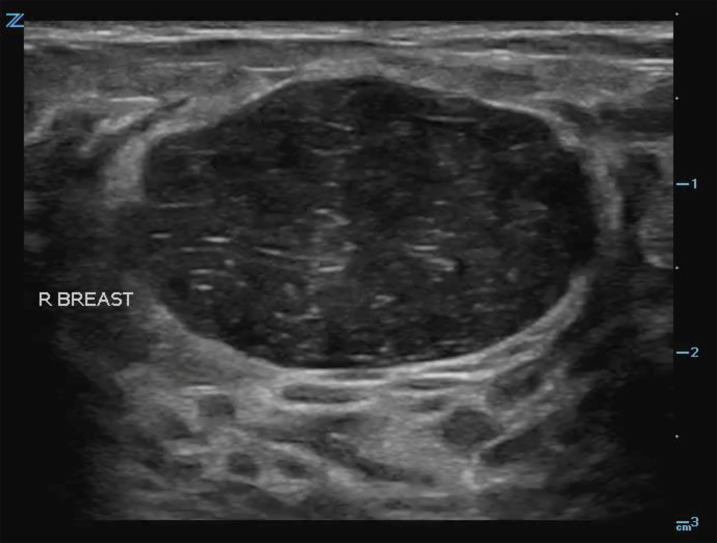
Solid, well-circumscribed mass found on a breast point-of-care ultrasound examination, later confirmed to be a fibroadenoma.

In several cases, the POCUS findings led EPs to obtain a surgical consult. Confirming a drainable fluid collection can prevent patients from undergoing painful and unnecessary procedures. For example, a young, female patient presented to the ED with a complaint of apparent breast swelling and a tender mass palpated near the areola. To further evaluate whether this was a soft tissue infection or a drainable fluid collection, the physician performed a POCUS examination and instead found a solid, well-circumscribed mass (Image 1). As these findings were more consistent with a fibroadenoma, an incision and drainage was not performed and antibiotics were withheld. The patient was sent to the breast clinic where the diagnosis of a fibroadenoma was confirmed. In some cases, POCUS was also used for needle guidance and to assess for successful drainage.

Only two of the patients in whom an abscess was suspected based on POCUS findings received additional imaging through radiology, which may speak to the confidence that these physicians had in diagnosing this particular breast pathology. In both of these cases, the physician’s original findings, which suggested a breast abscess, were confirmed with a radiology department breast ultrasound, and no new pathology was discovered. Findings were similar for patients in which cellulitis/mastitis was suspected based off a POCUS examination. The utility for POCUS in diagnosing skin and soft tissue infections is well documented.^14^,^15^ However, its role in the evaluation of skin and soft tissue infections in the breast has been significantly less explored. Forty-five percent of the patients were diagnosed with cellulitis or mastitis based off of a POCUS exam, and as a result all of these patients received a course of antibiotics. Of the two patients who had follow-up imaging, there were no discrepancies between the results of the studies. In regard to evaluating patients with breast complaints for abscess or skin and soft tissue infections, POCUS proved to be useful in guiding patient management.

**Image 2:**
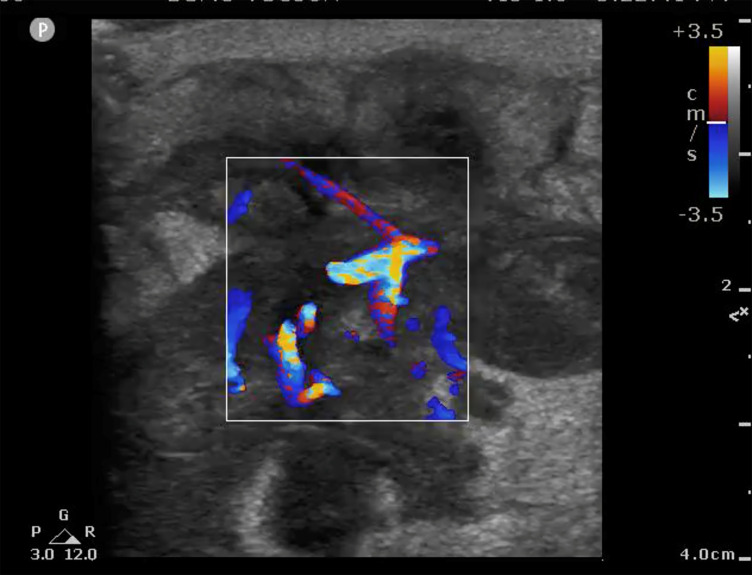
Irregular, highly vascularized structure found on a breast point-of-care ultrasound examination concerning for malignancy, later confirmed to be invasive ductal carcinoma.

Of greater importance perhaps is not finding a definitive diagnosis, but rather ruling out diagnoses that require urgent intervention and knowing when to suspect a more ominous process that requires an urgent follow-up or consultation with a specialist. For example, a female patient in this study presented initially to the ED after palpating a tender mass in her breast. The EP performed a breast POCUS examination at this visit, which demonstrated an irregular, highly vascularized structure concerning for malignancy (Image 2). Based on this imaging, the patient was secured an urgent follow-up appointment with the breast surgery clinic where she received a biopsy confirming invasive ductal carcinoma.

Patients who present to the ED with breast symptoms are often worried that the underlying cause is due to a malignant process. Although most breast concerns have benign, easily treatable causes, breast cancer is the most commonly diagnosed cancer among women and the second leading cause of cancer death in women in the United States.^16^,^17^ It is important that ED providers have an understanding of breast disease and have the ability to thoroughly evaluate and create an appropriate treatment plan.^18^ Mammography is the most commonly used modality for breast imaging, especially when screening for malignancy; however, it is not a study that is readily available in the acute care setting. In the ED, ultrasound is more readily available and better tolerated by the patient. But while breast ultrasound might be the mainstay for imaging in the evaluation of the breast in this setting, it is more often performed and interpreted by the department of radiology. This study is unique in that both the examination and interpretation were performed by the treating physician.

In this study, the characteristics of those patients presenting with breast complaints were recorded. Half of the patients who presented had a history of skin or soft tissue infection. All of these patients went on to have findings of a skin or soft tissue infection on POCUS examination. This also held true for the vast majority of diabetic patients. It was also documented whether patients were postpartum or lactating. This represents another important population as breast tissue undergoes significant physiologic changes during pregnancy and lactation. The benign physiological changes that occur during this time naturally lead to a denser parenchyma on imaging. These changes are seen sonographically with fibroglandular tissue that is of mixed echogenicity and disruption of the layered architecture. Emergency physicians may not be as familiar with theses findings on a breast ultrasound.

**Image 3:**
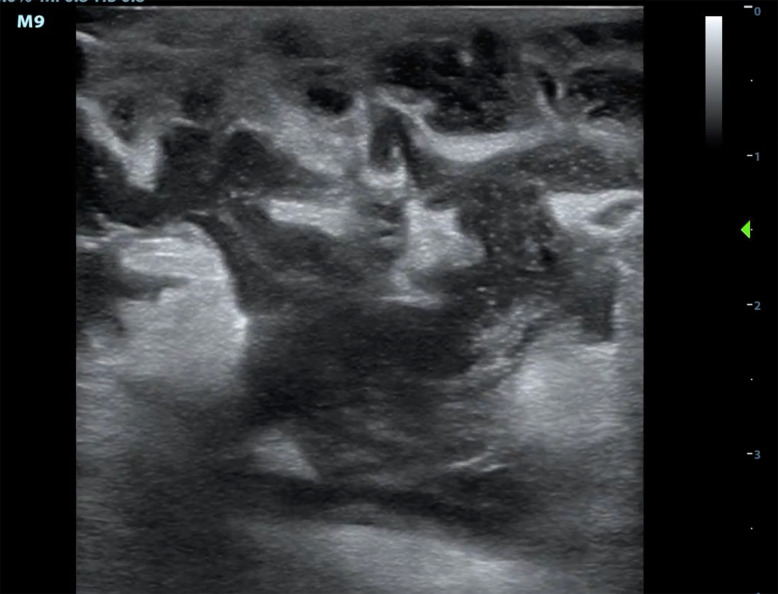
Breast point-of-care ultrasound examination showing dilated lactating ducts with surrounding abscess that required surgical consultation and drainage.

There are a number of benign, treatable findings commonly seen in postpartum and lactating patients that physicians should be aware of; these include galactoceles, lactating adenomas, mastitis, and abscesses (Image 3),^19^ all of which can be evaluated for in the ED using POCUS and guide further management. While approximately 80% of patients will have benign disease, it is important that EPs have the knowledge and ability to screen for more ominous processes in the ED setting.^20^–^23^ In our study, the breast POCUS examination findings in postpartum and lactating patients may have assisted in the medical decision-making process. Consultations were called on 83% of these patients. Several were admitted, with one requiring a same-day visit to the operating room.

Less common patient characteristics were also documented. Charts were reviewed to identify all patients with breast implants, as they may have presented unfamiliar challenges when evaluating for pathology. While there appear to be few studies that evaluate the long-term complications of breast augmentation, some indicate up to a 24% complication rate.^24^,^25^ The most common complication is pain but can also include infection and implant rupture. Prior studies have reported that ultrasound has a low sensitivity of 50–74% for detecting implant rupture, but suggest it may have a role as a screening modality.^25^–^27^ Half of the patients with breast implants who received a breast POCUS exam received a follow-up radiology-performed breast ultrasound. This made up a third of all patients who received follow-up radiology-performed studies in our study. suggesting that perhaps EPs are uncomfortable with performing and interpreting a breast POCUS examination in a patient with implants. However, these patients made up a small percentage (10%) of the patient population, making it difficult to draw any significant conclusions. Similarly, for pediatric and male patients, the sample sizes were too small to be conclusive.

## LIMITATIONS

This study has several limitations including its retrospective nature and the small sample size. This was not a multicenter study, which potentially limits the generalizability of our results. Another limitation of this study is the selection bias from the convenience sample design, since patients received a breast POCUS only when credentialed EPs were on duty. Additionally, it is likely that there were significantly more patient cases in which POCUS was used to assess a breast complaint, but images were not saved in the QPath database for reviewers to query. Therefore, we cannot say that this group accurately represented the full spectrum of patients presenting to the ED with breast complaints, limiting the generalizability of this study. The chart abstractor was not blinded to the study hypothesis and results; we attempted to reduce the bias in data collection by using a standardized data abstraction form.

Additionally, because there is also an emergency ultrasound fellowship program at this institution, the physicians are not only more experienced but also more driven to perform breast POCUS. Consequently, the practice patterns are not necessarily generalizable to the community ED. Further research showing the impact of breast POCUS is needed to support its application and fully realize its potential benefits on patient care in the ED. A prospective study design and further research that evaluates the diagnostic accuracy of breast POCUS for various pathologies would certainly add to the existing body of literature.

## CONCLUSION

Despite the limitations, our study findings illustrate the utility of point-of-care ultrasound in the evaluation of a variety of breast complaints in the ED. Our study suggests that breast POCUS has the potential to impact patient management in the diagnosis of pathology, in the performance of procedures, and in patient treatment and disposition.

## Figures and Tables

**Table 1:** Presenting symptoms of patients presenting to the emergency department with breast complaints.

Presenting Symptom	N (%)
Pain	23/40 (57.5)
Mass	15/40 (37.5)
Swelling	10/40 (25)
Redness	9/40 (23)
Cutaneous lesion	2/40 (5)
Discharge	2/40 (5)

**Table 2:** Characteristics of patients presenting with breast complaints.

Patient Characteristics	N (%)
History of skin/soft tissue infection	20/40 (50)
Surgical history	17/40 (42.5)
Diabetes Mellitus	6/40 (15)
Lactating	5/40 (12.5)
Postpartum	6/40 (15)
Breast implants	4/40 (10)
Male	4/40 (10)
Pediatric (<18 years of age)	3/40 (7.5)
History of breast cancer	2/40 (5)
Immunocompromised	1/40 (2.5)

**Table 3:** Breast point-of-care ultrasound findings.

Findings on POCUS	N (%)
Cobblestoning	20/40 (50)
Fluid collection (simple)	15/40 (37.5)
Increased tissue thickness	9/40 (22.5)
Fluid collection (complex)	8/40 (20)
Increased echogenicity	4/40 (10)
Hyperemia	3/40 (7.5)
No sonographic abnormalities	3/40 (7.5)
Positive “squish sign” (movement of echogenic particles in response to compression)	2/40 (5)
Homogenous mass	2/40 (5)
Heterogeneous mass	1/40 (2.5)

*POCUS*, point-of-care ultrasound.

**Table 4:** Emergency department diagnosis.

Diagnosis	N (%)
Abscess	14/40 (50)
Cellulitis	11/40 (27.5)
Mastitis	7/40 (17.5)
Breast mass	5/40 (12.5)
Breast pain	5/40 (12.5)
Lipoma	2/40 (5)
Breast wound	1/40 (2.5)
Fibroadenoma	1/40 (2.5)
